# Feasibility, Safety, and Tolerability of Remote Ischemic Conditioning in Children with Unilateral Cerebral Palsy: A Randomized Controlled Trial

**DOI:** 10.3390/children12101372

**Published:** 2025-10-11

**Authors:** Swati M. Surkar, Shailesh Gardas, John Willson, Joseph Kakyomya, Charity Moore Patterson

**Affiliations:** 1 Department of Physical Therapy, East Carolina University, Greenville, NC 27834, USA; gardass21@students.ecu.edu (S.G.); willsonj@ecu.edu (J.W.); 2 Department of Physical Therapy and School of Health and Rehabilitation Sciences Data Center, University of Pittsburgh, Pittsburgh, PA 15260, USA; jok145@pitt.edu (J.K.); cgp22@pitt.edu (C.M.P.)

**Keywords:** ischemic preconditioning, remote, cerebral palsy, child, rehabilitation, treatment outcome, randomized controlled trials

## Abstract

**Highlights:**

**What are the main findings?**

- Remote ischemic conditioning (RIC) was feasible in children with unilateral cerebral palsy (CP), with excellent recruitment, retention, and full adherence to the intervention protocol.
- Across more than 300 conditioning sessions, RIC was safe and well tolerated, with stable cardiopulmonary parameters and only minor, transient adverse events.

**What is the implication of the main finding?**

- RIC can be delivered reliably and safely in pediatric neurorehabilitation research, supporting its use in future large-scale clinical trials.
- These findings establish the groundwork for testing RIC as a novel priming strategy to augment neuroplasticity and functional recovery in children with CP.

**Abstract:**

Background: Remote ischemic conditioning (RIC) has shown promise as a neuroprotective strategy, but its application in children with cerebral palsy (CP) remains unexplored. We conducted a randomized controlled trial to evaluate the feasibility, safety, and tolerability of repeated, 6–7 sessions of RIC in children with unilateral CP. Methods: Fifty-one children aged 6–16 years with unilateral CP were randomized (1:1) to receive RIC or sham conditioning on the more affected arm. Primary feasibility outcomes included recruitment metrics, intervention adherence, retention, and protocol fidelity. Safety endpoints included continuous monitoring of oxygen saturation, blood pressure, heart rate, and adverse event incidence. Tolerability was assessed via child-reported pain ratings, conditioning pressure tolerance, skin integrity evaluations, and session adherence. Results: Of 148 children screened, 51 were randomized to RIC (n = 25), sham (n = 26) groups; 48 (94.1%) completed the intervention as allocated. Recruitment yielded 2.04 participants/month. Intervention adherence was 100% in both groups. RIC was well tolerated, with mean pain scores 2.8 ± 3.1 during inflation in RIC and 0.3 ± 0.8 in Sham group. No serious adverse events occurred. Physiological parameters remained stable across 314 conditioning sessions; no clinically significant hypoxemia, blood pressure derangements, or arrhythmias were detected. Minor adverse events (transient erythema, mild discomfort) were rare (2.22%) and self-limiting. Skin integrity was preserved, and no participants required session termination. Conclusions: Repeated RIC is feasible, safe, and tolerable in children with unilateral CP. These findings support the design of future trials using RIC as a priming agent to enhance pediatric neurorehabilitation outcomes.

## 1. Introduction

Unilateral cerebral palsy (CP) remains the most common subtype of childhood-onset motor disability, affecting approximately one-third of children diagnosed with CP [1]. Early brain injury often disrupts motor pathways controlling one side of the body, leading to persistent deficits in the more-affected upper limb [2]. Children with unilateral CP frequently experience impairments in hand control, coordination, and strength, leading to limitations in bimanual tasks essential for daily living, self-care, and participation [3,4]. These impairments persist despite therapy and impose lifelong barriers to independence, education, and social integration.

Intensive upper limb rehabilitation approaches such as constraint-induced movement therapy (CIMT) and bimanual training aim to improve function through task-specific, high-dose practice [5,6]. While these interventions have demonstrated efficacy, they often produce modest effect sizes with poor retention and require considerable time and effort to achieve clinically meaningful gains [7,8,9]. The limited responsiveness to therapy highlights a critical need to enhance the brain’s capacity for motor learning and plasticity. However, the effectiveness of existing rehabilitation approaches is limited, which underscores the need for a priming agent––an intervention delivered prior to training that can biologically prepare the nervous system to respond more effectively to rehabilitation.

Remote ischemic conditioning (RIC) may offer a promising solution to this gap. RIC is a non-invasive technique that involves brief, cyclic occlusion and reperfusion of blood flow in a limb (arm/leg) using a standard blood pressure cuff [10]. Originally developed to reduce myocardial infarction size during cardiac procedures [11], RIC has demonstrated systemic protective and adaptive effects across multiple organ systems, including the brain [12]. This technique induces sublethal ischemia at a remote site, triggering endogenous neuroprotective mechanisms [13,14]. Compared to direct ischemia at target organs, RIC presents a safer, more clinically feasible approach for inducing systemic preconditioning effects; hence, making it a compelling priming agent for integration into neurorehabilitation paradigms [15].

Mechanistically, RIC activates a cascade of neural, humoral, and metabolic responses that promote angiogenesis, neurogenesis, and synaptic plasticity [16,17]. Notably, RIC upregulates brain-derived neurotrophic factor (BDNF) [18], modulates GABAergic or inhibitory [19] and glutamatergic or excitatory activity [20], and improves cerebral perfusion [16]—all mechanisms known to support motor learning and experience-dependent plasticity [21]. Hence, RIC is increasingly conceptualized as a priming intervention, delivered prior to training to transiently enhance neural excitability, thereby augmenting subsequent task-specific practice. Early clinical trials support this concept and suggest that the neurobiological effects are amplified when RIC is paired with motor training [22,23]. In adults with stroke, RIC has been shown feasible, safe, and to augment motor recovery, increase muscle force in the paretic limb, and enhance neural activation when combined with task practice [24,25]. In healthy individuals, repeated RIC sessions improve upper limb performance metrics including muscular strength, endurance, and recovery [26,27,28].

Evidence also supports the safety and feasibility of RIC in pediatric populations. In children with moyamoya disease, RIC was safe, improved cerebral perfusion, and reduced ischemic events [29,30]. In neonates with necrotizing enterocolitis, RIC appeared to improve systemic inflammation and end-organ protection [31]. In neonates with encephalopathy, RIC was deemed safe and offered brain protection [32]. Collectively, these studies establish a favorable safety profile of RIC across both adult and pediatric populations, underscoring its translational potential. Despite this, its application in children with unilateral CP has not been systematically explored in the existing literature. This represents a critical and unaddressed knowledge gap, particularly given RIC’s capacity to biologically prime neural circuits and potentially amplify the effects of pediatric rehabilitation.

Despite robust evidence from adult stroke populations and encouraging safety and efficacy data in other pediatric conditions, no study has systematically investigated the feasibility, safety, and tolerability of RIC in children with unilateral CP. This absence of foundational data poses a significant barrier to translating RIC into pediatric neurorehabilitation frameworks. Prior to clinical adoption, researchers must rigorously evaluate RIC’s safety profile in this unique and vulnerable population. Establishing the tolerability and feasibility of repeated RIC administration is essential to ensure its integration as a viable adjunct to evidence-based upper limb therapies in unilateral CP.

## 2. Purpose of the Study

In this randomized controlled trial (RCT), we evaluated the feasibility, safety, and tolerability of repeated RIC in children with unilateral CP. We delivered RIC to the more affected arm and compared cardiovascular responses including heart rate, blood pressure, oxygen saturation and adverse events between RIC and sham groups. We also measured tolerability by tracking reported pain, conditioning pressure, bruising, adherence to sessions, and requests to terminate conditioning. Our findings lay the groundwork for future clinical trials that aim to combine RIC with upper limb rehabilitation to enhance functional outcomes in children with unilateral CP.

## 3. Materials and Methods

### 3.1. Study Design

This study was a single-center, prospective, double blind, RCT to primarily assess the effects of RIC combined with bimanual training on bimanual skill learning and corticospinal excitability in children with unilateral CP. The study protocol has been published elsewhere [33]. The ancillary data assessed the feasibility, safety, and tolerability of RIC in children with unilateral CP. The study was conducted in Pediatric Assessment and Rehabilitation Laboratory (PeARL), at East Carolina University (ECU).

### 3.2. Ethical Approval

The study was approved by ECU Institutional Review Board (approval number: UMCIRB 21-001913) and conducted between November 2022 and December 2024. The study was registered on clinicaltrials.gov (NCT05777070). The parents of participating children provided the written parental consent form, and the assent was obtained from children above 12 years. The study was performed in accordance with the ethical standards of Declaration of Helsinki.

### 3.3. Participants

The participating children had the diagnosis of unilateral CP and were recruited from various sources including local therapy clinics, the University of North Carolina Hospital’s Helping Kids with Hemiplegia program, Pitt County Public School Districts, referral from North Carolina school based physical therapists, therapy clinics from neighboring states of North Carolina, and Facebook groups for children with UCP. The inclusion criteria were: (1) children diagnosed with UCP between 6 and 16 years of age, (2) Manual Ability Classification System (MACS) levels I–III, (3) ability to complete a stack of three cups using two hands in one minute, ensuring a minimum threshold of bimanual ability required for engagement in the motor learning paradigm, and (4) mainstream in school and has sufficient cognition to follow the experimental procedures. Exclusion criteria were children with (1) other developmental disabilities such as autism, developmental coordination disorders, etc., as they may impact bimanual skill learning and affect trial results, (2) cognitive deficits or communication problem, (3) attention deficit hyperactive disorder (ADHD) or attention deficit disorders (ADD) as attentional difficulties can interfere with task performance and compliance with study procedures, (4) known cardiorespiratory and vascular dysfunction; sickle cell disease; cancer within the last 5 years or actively on anti-cancer drugs to ensure safety of RIC application, (5) actively receiving other adjunct therapies such as repetitive transcranial magnetic stimulation (rTMS), transcranial direct current stimulation (tDCS), vagus nerve stimulation, etc., as they may interfere with the RIC effects, (6) active seizures or a history of seizures in the past 2 years, (7) upper or lower extremity condition, injury, or surgery within last three months which could compromise conditioning and training. These criteria were established to maintain a relatively homogenous study population and to optimize our ability to determine conditioning safety and tolerability [33].

### 3.4. Sample Size and Randomization

The sample size was determined for the primary outcome of bimanual coordination, goal synchronization time as described in the published protocol [33]. A total of 40 participants (20 in each group) provided 80% power to detect an effect size of 0.90 at a significance level of 0.05. After accounting for a 25% drop out rate, we enrolled a total of 51 participants. The sample of 51 participants provided greater than 80% power to detect between group differences in all safety outcomes at a significance level of 0.05.

The study statistician generated a randomization schema in SAS V.9.4 using permuted blocks, which was subsequently uploaded into the REDCap (Vanderbilt University, Nashville, TN, USA) project randomization module to ensure allocation concealment. A stratified randomization method with age (below and above 10 years) and severity of hemiplegia based on MACS levels (mild = MACS level I, moderate = MACS levels II–III) was used to randomly assign the participants to the RIC or Sham conditioning groups. The participants and their families, and all outcome assessors remained masked to treatment allocation, and allocation concealment was preserved. Blinding was successfully maintained for the duration of the trial to minimize bias and ensure methodological rigor.

### 3.5. Intervention

Participants underwent a total of six to seven conditioning sessions, with the first six administered on consecutive days to the more affected arm, followed by a seventh session delivered within one week. More affected extremity was determined based on the subjective history and objective assessments of hand grip strength. During the first visit, we obtained demographic details and health and rehabilitation history. Pre-testing safety and tolerability measures were recorded, and the children were randomly assigned to the RIC or sham groups. Participating children were blinded to their group assignment until completion of all study visits. Trained research personnel, who were not blinded, performed the conditioning and obtained safety and tolerability measures. The assessors who were blinded to the group assignment analyzed the data.

### 3.6. Remote Ischemic Conditioning and Sham Conditioning Protocol

**Figure 1:**
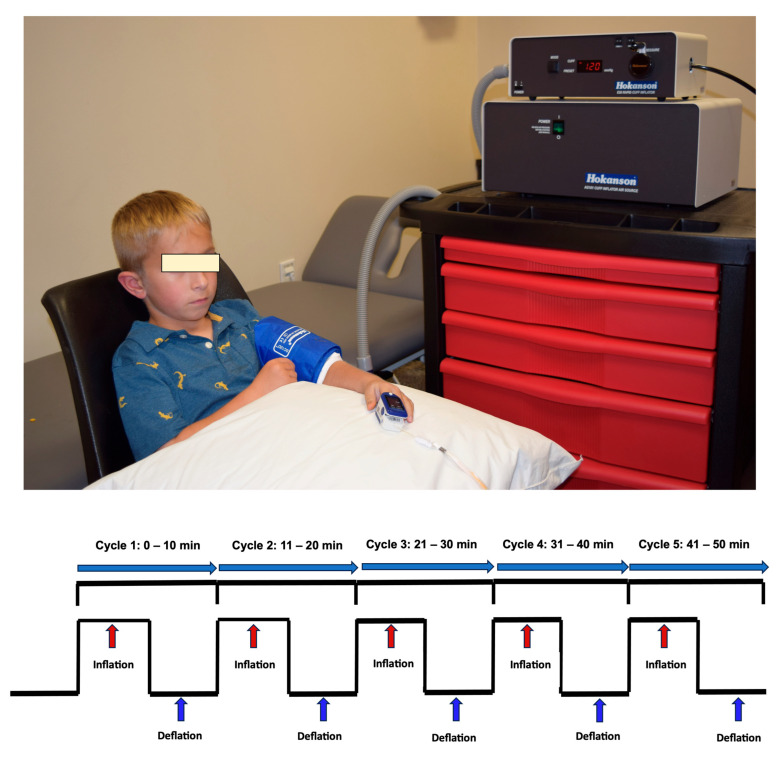
Remote Ischemic and Sham Conditioning Protocol.

Before beginning the conditioning, we recorded resting heart rate (HR), blood pressure (BP), oxygen (O_2_) saturation, skin integrity, and pain levels. Conditioning was performed by cyclic inflation and deflation of a pressure on the more affected arm (Figure 1) using a Hokanson rapid cuff inflator (D. E. Hokanson, Inc., Bellevue, WA, USA). Conditioning for the RIC and sham conditioning in the arm was delivered as a standard dose of 5 cycles of 5 min pressure cuff inflation followed by alternating 5 min of deflation resulting in a total conditioning time of 50 min. Children were allowed to engage in quiet, non-strenuous activities such as watching a movie, listening to an audiobook, or simply resting, and were instructed not to move their conditioning arm.

Consistent with previous studies, the conditioning pressure in the RIC group was >20 mmHg above that visit’s resting systolic BP since this pressure has shown as sufficient to induce ischemia and as effective as the standard 200 mmHg, with fewer side effects [23,26,34,35]. The conditioning pressure in the sham group was 25 mmHg since it gives the sensation of cuff inflation but does not induce arterial occlusion [36]. The experimenter continuously monitored the presence or absence of ischemia in the RIC and sham conditioning groups, respectively, by monitoring a pulse oximeter placed on the index finger of the conditioning arm and by visually inspecting the color of the conditioning arm. A reading of “0” for pulse and O_2_ saturation on the pulse oximeter and the presence of pale dusky appearance of the conditioning extremity confirmed the presence of ischemia in the RIC group. O_2_ saturation and pulse equivalent to baseline or prior to initiation of conditioning and unchanged color of the conditioning limb confirmed the absence of ischemia in the sham conditioning group. In the RIC group, if the pulse or O_2_ saturation reading appeared on the pulse oximeter anytime during the inflation cycle, the interventionist increased the inflation pressure until confirmation of ischemia on the conditioning arm, and the total time was adjusted to be consistent with 5 min of inflation cycle. The new pressure was used for further conditioning cycles. Similarly, in the sham conditioning group, if O_2_ saturation and pulse dropped below baseline measures, the conditioning pressure was decreased until preconditioning pulse and O_2_ saturation was achieved, and the arm showed no visible evidence of ischemia. Reperfusion during each 5 min deflation cycle was monitored in both groups and was confirmed by presence of O_2_ saturation and pulse rate returning to baseline level. In addition, O_2_ saturation was monitored on the non-conditioning limb to ensure cardiorespiratory stability.

For both groups, conditioning was immediately terminated if any of the following safety thresholds were met: (1) systolic BP < 85 mmHg or >140 mmHg; (2) diastolic BP < 50 mmHg or >90 mmHg; (3) HR < 60 bpm or >115 bpm; (4) O_2_ saturation < 90%; or (5) participant-reported pain score > 6. Additionally, conditioning was discontinued for that session if the absence of perfusion during the reperfusion cycle was observed on the conditioning arm through clinical inspection, including the lack of pulse restoration and failure of oxygenation levels to return to baseline.

On-site data verification was systematically performed throughout the study to ensure data integrity and protocol adherence.

Schematic illustration of the conditioning protocol administered to the more affected arm. Each session comprised five cycles of 5 min cuff inflation followed by 5 min deflation (total duration: 50 min), using a Hokanson rapid cuff inflator. In the RIC group, inflation pressure exceeded resting systolic blood pressure by >20 mmHg to induce transient ischemia; in the sham group, pressure was set at 25 mmHg to simulate inflation without vascular occlusion. Physiological parameters—heart rate, blood pressure, oxygen saturation, pain, and skin integrity—were assessed pre- and post-conditioning. During inflation, ischemia in the RIC group was confirmed by absent pulse and O_2_ saturation (reading of 0) on a fingertip pulse oximeter and dusky limb appearance of the conditioning limb; absence of ischemia in the sham group was verified by stable O_2_ saturation and limb color of the conditioning limb. Cuff pressure was adjusted as needed to maintain ischemic or non-ischemic states. Reperfusion during each deflation phase was confirmed by return of pulse and O_2_ saturation to baseline values. Cardiorespiratory stability was monitored throughout the conditioning using the non-conditioning limb.

### 3.7. Outcome Measures

#### 3.7.1. Feasibility Outcomes

Feasibility outcomes were defined a priori to evaluate the viability of the intervention and included recruitment metrics such as the number of participants contacted, enrollment rates, reasons for exclusion, recruitment efficiency, retention, attrition, and missing data. Conditioning adherence was quantified as the proportion of participants completed the protocol. Additional measures included fidelity, acceptability, which collectively informed the translational potential of the intervention.

#### 3.7.2. Safety Outcomes

The primary safety outcome assessed in this study was O_2_ saturation, while secondary safety outcomes included systolic and diastolic BP, HR, and the occurrence of adverse events (AEs) necessitating the discontinuation of conditioning. O_2_ saturation, BP, and HR were monitored on the non-conditioning arm using a pulse oximeter and digital BP monitors for both the RIC and Sham conditioning groups. Measurements were recorded at baseline, during, and immediately following each conditioning session to ensure participant safety and physiological stability.

All AEs, including seizures, were systematically documented during each conditioning session and within 24 h post-intervention. The severity of AEs was evaluated by trained research personnel, with serious adverse events (SAEs) assessed according to Institutional Review Board (IRB) guidelines. Treatment-emergent AEs were carefully monitored, including intolerance to RIC necessitating study withdrawal and any observable signs of tissue damage or neurovascular compromise attributed to the intervention. All AEs and SAEs were reported in accordance with regulatory and ethical oversight requirements to ensure participant safety and data integrity.

#### 3.7.3. Tolerability Outcomes

Tolerability of the intervention was primarily assessed through subjective pain perception in the conditioned limb, measured using a numeric pain scale (0–10), where 0 indicated no pain and 10 represented maximal discomfort [37]. Pain ratings were systematically recorded at the end of each 5 min cuff inflation within the conditioning cycle to capture peak discomfort, with average pain during conditioning reported. Secondary tolerability measures included the average conditioning pressure applied throughout the study, skin integrity assessments (monitoring for bruising, wounds, skin excoriations, petechiae, or hematomas) at multiple time points, and adherence to the conditioning protocol, defined by the number of participant-initiated requests to terminate conditioning and the total number of conditioning sessions completed. These measures provided a comprehensive evaluation of the intervention’s tolerability, ensuring the assessment of both physiological and behavioral responses to the conditioning protocol.

### 3.8. Statistical Analysis

All statistical analyses were performed using SAS (version 9.4). Analyses were conducted in accordance with the intention-to-treat principle with all randomized participants included except one who was excluded due to protocol contamination. All tests were two-sided, and a *p*-value of less than 0.05 was considered statistically significant. Data distributions were examined for normality using Q-Q plots and Shapiro–Wilk test. When assumption of normality was satisfied, parametric tests were applied; otherwise, appropriate non-parametric tests were used.

#### 3.8.1. Feasibility Outcomes

Feasibility outcomes were defined a priori to evaluate the viability of the intervention and included recruitment metrics such as the number of participants contacted, enrollment rates, reasons for exclusion, recruitment efficiency, retention, attrition, and missing data. Conditioning adherence was quantified as the proportion of participants completed the protocol.

Additional measures included fidelity, acceptability, which collectively informed the translational potential of the intervention. Fidelity assessments were based on standardized session logs completed by trained interventionists, capturing inflation pressure, cycle duration, total number of completed cycles, and any protocol deviations. Participant engagement and acceptability of the RIC or sham conditioning protocols were evaluated through child self-reports, observational metrics documented by interventionists, and caregiver surveys during and after each session. These measures captured indicators such as willingness to participate, perceived burden, pain tolerance, behavioral compliance, and subjective satisfaction with the intervention experience.

#### 3.8.2. Safety Outcomes

Safety outcomes were analyzed using linear mixed-effects models, with groups (RIC vs. Sham) as between subject factor and time (pre, during, and post-conditioning) as the within-subject factor. Sphericity was assessed via Mauchly’s test; where violated, Greenhouse–Geisser corrections were applied. To evaluate between-group differences at each time point, contrasts were used.

AEs and SAEs were summarized descriptively. AEs were coded by severity and relation to the intervention (RIC vs. Sham) and reported according to IRB guidelines. Due to low event frequency, inferential testing was not conducted.

#### 3.8.3. Tolerability Outcomes

Tolerability outcomes were evaluated using both within and between-session analyses. To assess changes in pain scores across sessions, linear mixed model was applied with session as a repeated measure and group as a fixed effect. Average pain scores (Numeric Pain Rating Scale: 0–10) across seven sessions were compared between groups using independent-samples *t*-tests, with effect sizes reported using Cohen’s *d* and corresponding 95% CIs. The average conditioning pressure across sessions were analyzed similarly. Additional secondary tolerability endpoints—including the frequency of participant-initiated termination requests and dermatologic integrity (e.g., petechiae, bruising, excoriation)—were summarized descriptively.

All missing data were addressed using restricted maximum likelihood estimation (REML) within the mixed model framework. Complete case analyses were also conducted to confirm result robustness.

## 4. Results

Table 1 provides a detailed summary of the baseline demographic and clinical characteristics for the 51 participants randomized into either the RIC or Sham conditioning groups. No significant differences in baseline characteristics were observed between the treatment groups.

### 4.1. Feasibility Outcomes

**Recruitment and Enrollment Metrics**

Between November 2022 and December 2024, 321 children diagnosed with unilateral CP were identified and contacted for potential participation in the trial. Of these, 173 (53.9%) did not respond to initial recruitment outreach. The remaining 148 children (46.1%) underwent a formal eligibility assessment based on pre-specified inclusion and exclusion criteria.

A total of 97 children (65.5% of those screened) were excluded following eligibility screening. Of these, 85 children were deemed ineligible due to not meeting one or more predefined inclusion criteria. The most frequent reasons for exclusion included active seizures or history of seizures (n = 49), attention deficit disorder (ADD)/attention-deficit/hyperactivity disorder (ADHD) (n = 7), out-of-range age (n = 6), inability to complete the cup-stacking task used to assess motor function (n = 7), recent botulinum toxin injections within the prior 6 months (n = 3), presence of hydrocephalus requiring a shunt (n = 3), cochlear implant (n = 2), cardiovascular comorbidities (n = 2), active or prior neoplasm (n = 2), metal implants related to arteriovenous malformation (AVM) coiling (n = 2), and coexisting cognitive dysfunction and other neurological disorders beyond unilateral CP (n = 2).

An additional 12 children were excluded after meeting eligibility criteria but choosing not to participate. Among these, 3 declined for personal reasons, 8 cited logistical barriers such as travel cost and time commitment, and 1 was excluded at the discretion of their neurologist due to concern about the risk of triggering prior seizures.

**Figure 2:**
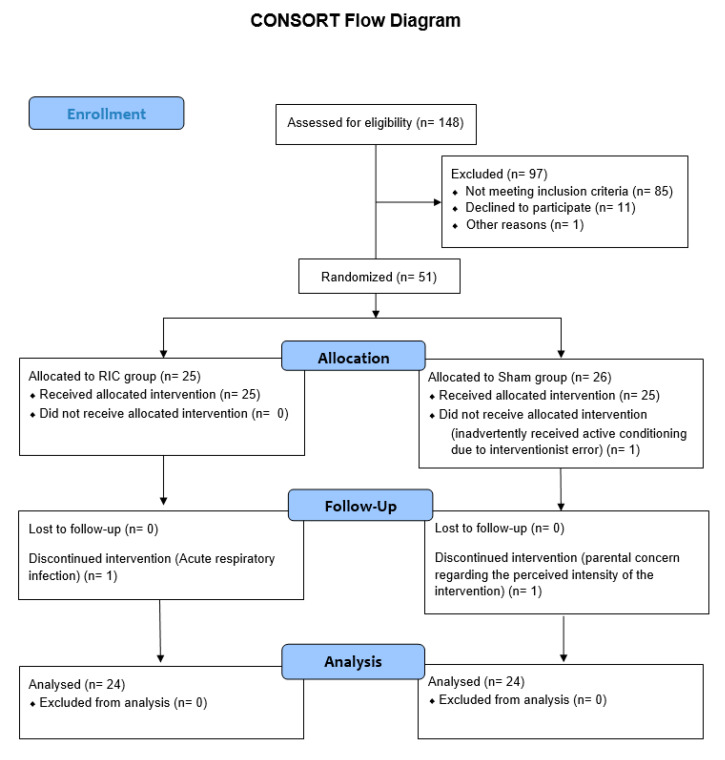
CONSORT Flow Diagram of Participant Progress Through the Study. Of 148 individuals assessed for eligibility, 97 were excluded (85 did not meet inclusion criteria, 11 declined to participate, and 1 was excluded for other reason). A total of 51 participants were randomized to receive either remote ischemic conditioning (RIC; n = 25) or sham conditioning (n = 26). All participants in the RIC group received the allocated intervention. In the sham group, 1 participant inadvertently received active conditioning due to an interventionist error in applying a higher-than-intended pressure during the sham procedure. During the intervention period, 1 participant in the RIC group discontinued participation due to an acute respiratory infection, and 1 participant in the sham group discontinued due to parental concern regarding the perceived higher intensity of the intervention. No participants were lost to follow-up. A total of 48 participants (RIC: n = 24; Sham: n = 24) completed the study and were included in the final analysis. No participants were excluded from analysis.

Fifty-one children (31.4% of those screened) were enrolled and randomized in a 1:1 allocation to receive either (RIC; n = 25) or sham conditioning (n = 26). Participants’ flow through the trial is shown in the CONSORT diagram (Figure 2).

Of the 51 randomized participants, 48 (94.11%) completed the intervention protocol as allocated. Reasons for drop out during intervention phase included an acute respiratory tract infection (n = 1), incongruence between family capacity and intervention demands (n = 1). Additionally, one participant was excluded from the intention-to-treat analysis due to the protocol deviation where participant inadvertently received active conditioning due to interventionist error in applying a higher-than-intended pressure during the sham conditioning procedure (n = 1). As a result, 48 participants were included in the analysis of outcome measures.

### 4.2. Recruitment Efficiency and Retention

Recruitment efforts yielded a consistent enrollment rate across the 25-month period, averaging approximately 2.04 participants per month. Retention was excellent, with 94.4% of enrolled participants completing all scheduled assessments for intention-to-treat analysis. The high retention indicates robust participant engagement and procedural acceptability.

### 4.3. Attrition

Attrition in the trial was minimal. Of the 51 participants randomized, two (3.92%) did not complete the entire study protocol. One participant in the RIC group (1.96%) discontinued after a single conditioning session due to an acute respiratory illness, while another in the sham group (1.96%) withdrew secondary to incongruence between the family capacity and the intervention’s physical demands. Across the entire dataset, session-level data were complete for all participants with the exception of a single session for which documentation was unavailable due to a missing session log, representing <0.5% of total session data. These findings indicate excellent participant retention and data completeness throughout the intervention period.

**Adherence**

Adherence to the intervention protocol was high across both study arms. Of the 51 randomized participants, 48 (94.1%) completed the intervention as allocated. Participants were scheduled for six conditioning sessions, with a subset (n = 29) receiving a seventh on the day of post-assessment to maintain conditioning effects. No participants discontinued the intervention due to AEs, intolerance, or personal request. Across all administered sessions, the median adherence rate was 100% reflecting high procedural fidelity and participant engagement. These findings underscore the feasibility and acceptability of repeated RIC administration in a pediatric clinical trial setting.

**Intervention Fidelity**

Of the 314 conditioning sessions, 307 (97.7%) were delivered in accordance with the study protocol. Of the seven sessions not delivered with full fidelity, six sessions (2.4%) from a single participant were affected by protocol contamination, and one session (0.3%) was impacted by a missing documentation sheet.

All interventionists received structured training, and protocol implementation was regularly overseen by the principal investigator to maintain procedural consistency. However, one instance of cross-contamination was identified, wherein a participant randomized to the sham group inadvertently received active RIC due to application of a higher-than-intended inflation pressure. This protocol deviation was identified, and the participant was excluded from the intention-to-treat analysis as a pre-determined criteria to preserve the integrity of group allocation.

No additional deviations or breaches in randomization or blinding were reported, and all other sessions were delivered with high procedural fidelity. These findings support the reliability and internal validity of the intervention delivery across study arms.

**Acceptability and engagement**

Across both study groups, children demonstrated consistent willingness to participate, with interventionists reporting high compliance (97.3%) in limb positioning and tolerance of cuff inflation during conditioning sessions. Engagement remained stable across repeated sessions, and no participant requested early termination due to pain or adverse events. Pain ratings, assessed with Numeric Pain Rating Scale, were consistently within the mild to moderate range (0–5), with no reports of severe pain (>6) requiring discontinuation. One participant withdrew after the first session due to parental concerns regarding intervention intensity, which was not related to pain or adverse events.

Caregiver survey responses further supported acceptability with 88% caregivers rated the intervention as “easy to follow” and 86.4% reported it as “time-efficient”. Thematic analysis of open-ended survey responses highlighted positive perceptions of engagement, perceived benefits, and acceptable within the context of daily routine.

### 4.4. Safety Outcomes

#### 4.4.1. Oxygen Saturation

**Figure 3:**
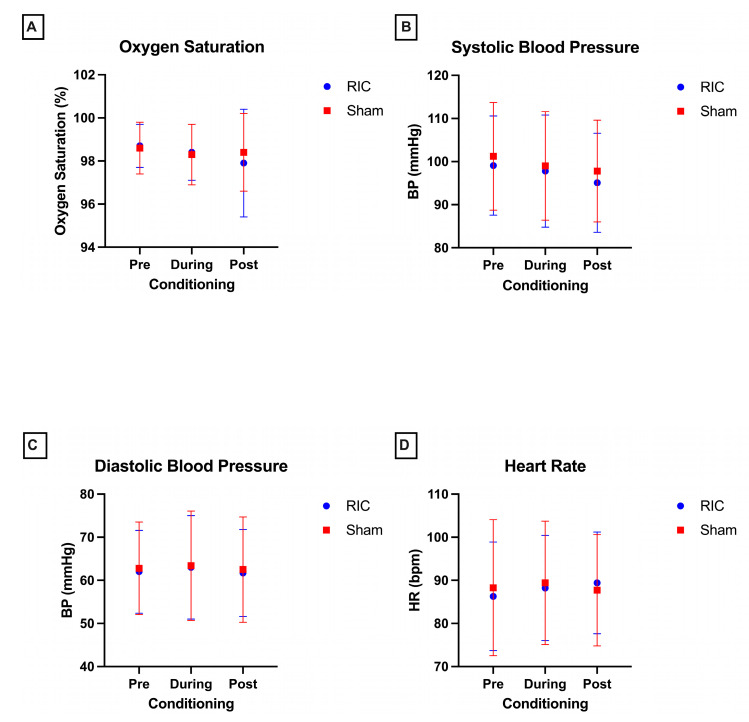
Cardiopulmonary safety profiles during remote ischemic conditioning (RIC) and sham conditioning. (**A**) Peripheral oxygen saturation remained stable, with mean SpO_2_ values consistently within physiological limits and no episodes of hypoxemia (SpO_2_ < 90%) in either group. (**B**,**C**) Systolic and diastolic blood pressure trajectories demonstrated no significant intragroup or intergroup variability, and no participant exceeded age- and sex-adjusted thresholds for hypertension or hypotension. (**D**) Heart rate monitoring revealed physiologically consistent temporal trends across baseline, conditioning, and recovery, with no bradycardic or tachycardic responses outside age-appropriate reference ranges. Data are presented as mean ± SD.

O_2_ saturation, the primary safety endpoint, remained within physiologically normal limits across both intervention arms. Continuous monitoring on the non-conditioning limb revealed no clinically significant hypoxemia (SpO_2_ < 90%) during or after conditioning. There was no significant main effect of group (*p* = 0.60); however, there was a significant group x time interaction (*p* = 0.026). The post hoc analysis revealed a modest difference in post-conditioning values between RIC (97.9 ± 2.5%) and sham (98.4 ± 1.8%) groups (*p* = 0.012); however, this variation was physiologically negligible and clinically non-significant. Overall, the results indicate stable values across pre-, during, or post-conditioning in both the RIC and sham groups (Figure 3A). Mean O_2_ saturation during conditioning was 98.4% ± 1.3% in the RIC group and 98.3% ± 1.4% in the sham group. No participants required supplemental oxygen or medical intervention for oxygen desaturation at any point.

#### 4.4.2. Blood Pressure

Systolic and diastolic BP remained stable throughout all study phases. There was no significant effect of group or group × time interaction for either systolic or diastolic trajectories (all *p* > 0·05), indicating comparable patterns across baseline, conditioning, and recovery in both RIC and sham groups (Figure 3B,C). Mean systolic BP during conditioning was 97.8 ± 13 mmHg in the RIC group and 99 ± 12.60 mmHg in the sham group, while mean diastolic BP values were 63 ± 12 mmHg and 63.4 ± 12.7 mmHg, respectively. No participant experienced hypertensive (>95th percentile for age and sex) or hypotensive (<5th percentile) responses necessitating discontinuation of conditioning.

#### 4.4.3. Heart Rate

HR monitoring similarly revealed no aberrant cardiovascular responses attributable to the intervention. There were no significant effects of group (*p* = 0.64) or group × time (*p* = 0.07) interaction. The mean HR during conditioning was 88.0 ± 12.21 bpm in the RIC group and 89.4 ± 14.3 bpm in the sham group, with no participant exhibiting sustained bradycardia or tachycardia outside the age-appropriate reference ranges. Temporal trends across the conditioning cycle were physiologically consistent between both groups (Figure 3D).

#### 4.4.4. Adverse Events

No serious adverse events (SAEs) occurred during the intervention period. Eleven minor AEs were documented in the RIC group, including transient erythema at the conditioning site (n = 7) and mild discomfort such as tingling, numbness or tightness during cuff inflation (n = 4); both resolved within 24 h and did not necessitate clinical intervention. No AEs led to discontinuation of treatment or affected data integrity.

Importantly, no seizure activity was observed in any participant during or following the intervention. Post-session monitoring within 24 h also revealed no delayed AEs or signs of neurovascular compromise.

#### 4.4.5. Tolerability Outcomes

**Subjective Pain Perception**

**Figure 4:**
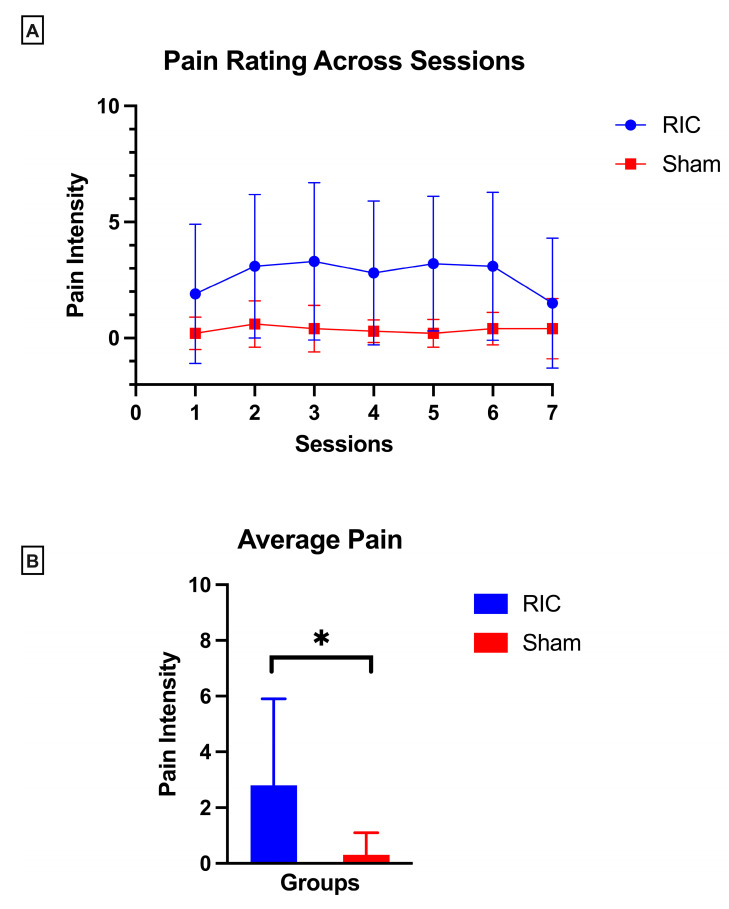
Pain perception during remote ischemic conditioning (RIC) and sham conditioning. (**A**) Longitudinal pain ratings across seven conditioning sessions remained stable within each group, with no evidence of sensitization or progressive discomfort over time. (**B**) Between group differences in average pain rating during conditioning. Between-group comparisons demonstrated higher average pain scores in the RIC compared to sham group. Data are presented as mean ± SD; asterisk (*) denotes statistical significance.

Longitudinal analysis across all seven sessions demonstrated that pain ratings during conditioning remained stable in both groups, with no evidence of sensitization or progressive increase in discomfort over time (Group × Session interaction: RIC: *p* = 0.49, Sham: *p* = 0.66) (Figure 4A).

In the RIC group, average pain scores remained minimal prior to each session (median 0, IQR 0–0), increased transiently during cuff inflation (mean 2.8 ± 3.1), and consistently returned to baseline immediately post-conditioning (median 0, IQR 0–0). The sham group reported negligible pain at all time points (pre-session, during, and post-session median scores all 0, with intra-session mean 0.3 ± 0.8). No participant in either group reported pain exceeding a score of 6 at any time point.

Between-group comparisons revealed a statistically significant difference in pain perception during conditioning (*p* = 0.001, *d* = 1.1), with the RIC group reporting higher scores relative to the sham group (Figure 4B). These findings support the intervention’s strong tolerability, reinforce the absence of cumulative pain burden and support the behavioral tolerability of repeated RIC administration.

**Conditioning Pressure**

Conditioning pressure remained stable across all seven sessions in both RIC and sham groups, with minimal between-session variability (RIC: *p =* 0.06, sham: *p* = 0.05).

**Figure 5:**
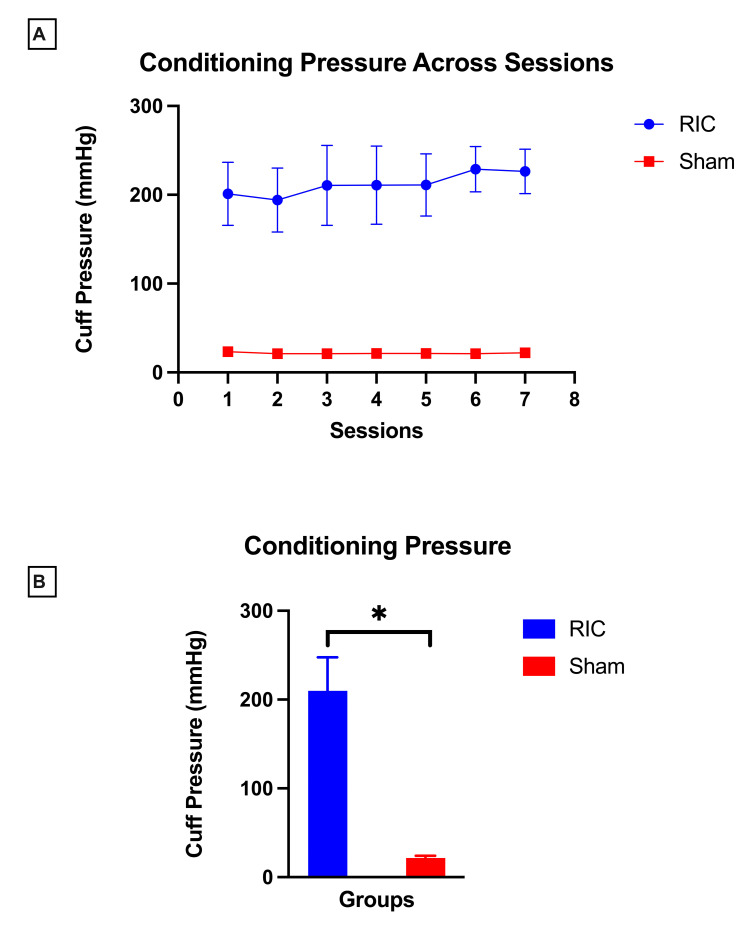
Conditioning pressure during remote ischemic conditioning (RIC) and sham conditioning. (**A**) Conditioning pressures remained stable across all seven sessions within each group, with higher conditioning pressures in the RIC group and lower pressures in the sham group. (**B**) Between-group differences in average conditioning pressure. Pressures were significantly higher in the RIC compared to sham group, but all were well tolerated. Data are presented as mean ± SD; asterisk (*) denotes statistical significance.

Participants in the RIC group received occlusive pressures calibrated individually based on systolic BP and evidence of ischemia throughout the conditioning cycles, with a study-wide mean conditioning pressure of 209.8 ± 37.9 mmHg. In contrast, participants in the sham group received non-occlusive pressures, with a mean value of 21.6 ± 2.3 mmHg across conditioning sessions. There was a significant between-group difference (*p* = 0.001, *d* = 7) in conditioning pressure, with RIC group requiring greater pressure compared to sham group (Figure 5B).

**Skin Integrity Monitoring**

Skin integrity was evaluated through visual inspection of the conditioned limb before and after each session by trained personnel. Examiners documented any evidence of bruising, petechiae, abrasions, hematomas, or discoloration and used clinical judgement to determine whether findings were of concern. Across the entire cohort (N = 51), no participant exhibited clinically concerning dermatologic reactions. While visual inspection is limited in its ability to detect subclinical changes, these findings support the non-invasive nature of the procedure and absence of cutaneous trauma associated with conditioning.

**Participant-Initiated Terminations and Protocol Adherence**

Tolerability was supported by full session adherence: none of the 48 participants who completed the intervention requested early termination, and all sessions were completed as prescribed in both the RIC and sham groups.

Collectively, these metrics affirm the high tolerability profile of the RIC protocol in pediatric participants with unilateral CP, without compromising procedural adherence or safety.

## 5. Discussion

This prospective, randomized controlled trial constitutes the first rigorous evaluation of the feasibility, safety, and tolerability of RIC in children with unilateral CP, addressing a critical gap in the translational advancement of non-invasive priming strategies for pediatric neurorehabilitation. Our findings demonstrate that RIC is not only safe and feasible but also well tolerated in this population, thereby establishing a foundational framework for its incorporation into adjunctive rehabilitation protocols. The robustness of these conclusions is supported by high recruitment efficiency, excellent protocol adherence, minimal attrition, and the absence of SAEs across 314 conditioning sessions. Importantly, this study demonstrates that children with CP, who are often underrepresented in early-phase interventional trials due to heightened safety concerns and anticipated participant burden, can successfully participate in and tolerate RIC. Hence, these findings highlight the timely and transformative potential of RIC as a novel adjunct to enhance neurorehabilitation outcomes.

### 5.1. Feasibility: Setting a Precedent for Neurorehabilitation Trials in Pediatric Populations

This feasibility trial demonstrates that RIC is logistically and procedurally feasible, and behaviorally acceptable in children with unilateral CP. The high rates of protocol adherence, participant retention, and procedural fidelity reflect the remarkable engagement of children and their caregivers. The recruitment of 51 participants across a 25-month period despite a highly selective inclusion process underscores both the clinical interest in innovative non-invasive interventions and the challenges inherent in targeting a heterogeneous pediatric population with complex neurological needs. Importantly, the reasons for ineligibility, dominated by seizure history and comorbid neurodevelopmental conditions such as ADHD, highlight the need for broader safety data and perhaps alternative trial designs that are inclusive of these common co-occurring conditions. Attrition was minimal and retention of participants throughout the study protocol was exemplary with 94.1% participants completing the entire study protocol and a median adherence rate of 100% across both groups. The structured, non-invasive nature of RIC along with its brief session duration and integration within a familiar therapeutic milieu likely contributed to the low dropout rate (3.9%). This level of compliance in a repeated intervention protocol is especially noteworthy in a pediatric cohort and suggests that RIC is intuitively manageable within the context of routine care. This robust feasibility also contrasts with historical challenges associated with pediatric neurorehabilitation trials, in which recruitment and retention are frequently impeded by logistical complexities, concerns surrounding sham randomization, and the risk of adverse effects—factors that have traditionally constrained the implementation of such trials [38,39,40].

The study also demonstrates high intervention fidelity, with near-complete protocol execution across all sessions and only one instance of cross-contamination. These data support the internal validity of the trial and reflect the rigor of both interventionist training and oversight mechanisms. The meticulous capture of fidelity metrics also strengthens the translational potential of this intervention, by modeling real-world implementation pathways. One of the most compelling findings pertains to the behavioral and psychosocial acceptability of RIC. Despite its counterintuitive biological mechanism, temporarily restricting blood flow to induce systemic neuroprotection, children demonstrated sustained engagement and willingness to participate across multiple sessions, with minimal signs of distress, fatigue, or behavioral disengagement. Caregivers likewise reported a high degree of satisfaction, noting the intervention’s compatibility with family routines, which are crucial determinants of successful translation from controlled trials to real-world rehabilitation settings. Taken together, these findings suggest that RIC meets the essential benchmarks of feasibility in children with unilateral CP. Importantly, the success of this trial aligns with emerging evidence from adult stroke populations, where RIC has demonstrated high feasibility even in acute care settings [24,41,42]. Furthermore, preliminary pediatric work has supported the feasibility of RIC in neonatal encephalopathy and in pediatric moyamoya disease [29,32].

### 5.2. Safety: Rethinking the Risk Threshold in Neurotherapeutic Innovation

Safety profiles in pediatric interventions must be held to the highest standard, particularly when involving novel conditioning stimuli applied repetitively. Our findings provide compelling evidence that RIC is safe in a pediatric neurorehabilitation context, establishing a crucial foundation for future efficacy trials targeting motor recovery and neuroplasticity. Physiological monitoring across 314 sessions revealed no clinically significant perturbations in O_2_ saturation, BP, or HR, attesting to the intervention’s cardiovascular stability. O_2_ saturation remained consistently within normative thresholds, with no episodes of desaturation necessitating intervention. Similarly, systolic and diastolic BP trajectories were remarkably stable across conditioning cycles, with no hypertensive or hypotensive events. HR responses were within typical range indicative of cardiovascular safety. These safety findings are consistent with adult data where repeated RIC exposures are well tolerated, with no adverse effects on hemodynamics or end-organ function, even in populations with vascular compromise [43,44]. Moreover, recent pediatric trials in moyamoya disease, neonatal encephalopathy and necrotizing enterocolitis report similar safety profiles, despite higher medical vulnerability [30,31,32,45].

Importantly, no SAEs were observed. The minor AE reported (transient erythema and mild cuff-related discomfort) were self-limiting, clinically inconsequential, and consistent with prior RIC trials [30,41,43,44]. Crucially, the absence of seizure activity, a paramount safety concern in pediatric populations with neurodevelopmental disorders, further supports the neurological safety of RIC when administered under stringent clinical protocols.

Taken together, these data strongly endorse the physiological tolerability of repeated limb ischemia–reperfusion cycles in children with unilateral CP, an observation that carries profound translational implications. RIC, by virtue of its non-invasive nature, low resource demands, and excellent safety profile, emerges as a highly attractive candidate for integration into pediatric neurorehabilitation paradigms.

### 5.3. Tolerability: Aligning Intervention Demands with Developmental Realities

Our study demonstrated that RIC is tolerable in children with unilateral CP, with tolerability evaluated across pain perception, conditioning pressure tolerance, skin integrity, and adherence. Self-reported pain scores, a cornerstone of patient-centered tolerability assessment, remained low across the intervention, with no reports of severe discomfort and no temporal sensitization across repeated exposures. Notably, the transient, modest pain elevations during cuff inflation resolved immediately post-session, indicating nociceptive stimulus was fleeting and manageable. Importantly, no participant exceeded a pain rating of 6 in RIC group, and no attrition due to discomfort was observed, highlighting the intervention’s acceptability even among pediatric cohorts with impaired somatosensory processing.

Conditioning pressures were calibrated meticulously to individual limb occlusion thresholds, achieving robust ischemic stimuli without compromising comfort or limb integrity. Median pressures of 180 mmHg, while physiologically significant, were consistently well-tolerated, attesting to the feasibility of delivering clinically meaningful ischemic loads without precipitating distress. Sham procedures, employing non-occlusive pressures, further validated the specificity of observed tolerability patterns. Importantly, while the study protocol defined conditioning pressure as >20 mmHg above systolic BP, the actual pressure required to elicit and sustain limb ischemia was significantly higher resulting in a median conditioning pressure of 180 mmHg. This value, although within the literature-supported range of 20 mmHg above systolic BP up to 250 mmHg, raises important considerations for future clinical trials in pediatric populations with CP [34,46,47]. The need for higher pressures may reflect altered peripheral physiology in this population, potentially due to increased muscle tone (spasticity), vascular remodeling, or microcirculatory changes associated with neurodevelopmental injury [48,49]. These findings underscore the importance of individualized pressure titration to balance efficacy and safety in future dose–response studies.

Our study also supports dermatological safety with RIC and no cases of skin breakdown, bruising, petechiae, or neurovascular compromise were documented. Preservation of skin integrity across all sessions emphasizes RIC’s non-invasive nature.

Notably, full protocol adherence was achieved, with no early session terminations, highlighting both the physiological and psychological acceptability of RIC. In pediatric rehabilitation research, where engagement and compliance often represent substantial challenges, such adherence is both remarkable and clinically meaningful. It reflects not only the physiological tolerability of RIC but also its psychological acceptability to young participants and their families. Clinically, these findings bear significant translational implications–– while RIC requires individualized pressure titration, the procedure can be delivered by trained personnel following a structured protocol, without the need for advanced clinical judgement or complex monitoring systems. Standardized training and competency checks would be sufficient for safe administration in both clinical trials and future clinical care.

### 5.4. Translation to Mechanistic and Efficacy Trials

The translational significance of these findings is multifold. First, the intervention’s excellent safety and tolerability profile positions RIC as a promising priming agent that could be layered onto conventional therapies without disrupting existing care pathways. Second, its non-invasive, low-cost nature makes it particularly attractive for low-resource settings or populations with limited access to intensive rehabilitative services. Third, the engagement and adherence metrics observed here support the feasibility of longer-term administration, which may be critical for achieving durable neuroplastic gains in children with evolving motor systems.

From a mechanistic standpoint, in preclinical and early clinical models RIC has been shown to activate pathways that reduce acute cerebral injury and promote plasticity, including neurotrophic signaling, angiogenesis, and excitatory–inhibitory balance [16,18,19,20]. These mechanisms align with the targets of neurorehabilitation in children with CP. Establishing feasibility and safety in this trial sets the stage for subsequent efficacy studies powered to detect changes in motor outcomes, cortical reorganization, and neurovascular coupling.

## 6. Limitations

Several limitations warrant acknowledgment. First, the study’s exclusion of children with active seizure disorders and other common comorbidities such as ADHD, although justified for initial safety evaluation, may limit generalizability. Subsequent investigations should explore the applicability and safety of RIC in more diverse and representative pediatric neurorehabilitation populations, particularly given the high prevalence of multisystem involvement in children with CP. Second, while subjective pain reports and skin assessments captured key domains of tolerability, additional multidimensional measures including psychophysiological stress markers, neurovascular imaging, and child-reported affective responses could enrich understanding of the full biopsychosocial impact of repeated ischemic conditioning. Third, the intervention was delivered in a structured clinical setting with close monitoring, which may not fully reflect real-world implementation challenges. Pragmatic trials assessing RIC integration into standard outpatient or community-based rehabilitation programs will be essential to validate feasibility and adherence outside research environments. Finally, the neurobiological mechanisms underlying RIC’s potential benefits in children with CP remain speculative, extrapolated primarily from adult and preclinical models. Future studies incorporating mechanistic endpoints such as serum biomarkers of neurotrophic signaling, neuroimaging of cortical reorganization, and electrophysiological indices of corticospinal excitability are critical to elucidate the biological substrates of RIC-induced neuroplasticity in the developing brain.

## 7. Conclusions

In conclusion, this trial provides the first robust evidence that repeated administration of RIC is feasible, safe, and well-tolerated in children with unilateral CP. These results have substantial translational implications, paving the way for the integration of RIC into multi-modal rehabilitation strategies aimed at enhancing motor recovery and neuroplasticity in pediatric populations. Future studies should explore the neurophysiological mechanisms underpinning RIC’s potential benefits and examine its synergistic effects when combined with task-specific motor training.

## Figures and Tables

**Table 1:** Baseline demographic and clinical characteristics of participants.

Characteristic	RIC Group (n = 25)	Sham Group (n = 26)
**Age**, years mean (SD), [range]	10.8 (2.9), [7–16]	9.4 (3.3), [6–16]
**Sex**		
Male	17 (68.0%)	23 (88.5%)
Female	8 (32.0%)	3 (11.5%)
**Ethnicity**		
Hispanic/Latino	2 (8.0%)	4 (15.4%)
Not Hispanic/Latino	23 (92.0%)	22 (84.6%)
**Race**		
Asian	3 (12.0%)	2 (7.7%)
Black/African American	2 (8.0%)	2 (7.7%)
White	20 (80.0%)	19 (73.1%)
Unknown/Not reported	0	2 (7.7%)
Missing	0	1 (3.8%)
**Severity of cerebral palsy**		
Hemiplegia	24 (96.0%)	25 (96.2%)
Quadriplegia	1 (4.0%)	1 (3.8%)
**MACS Levels**		
I	2 (8%)	4 (15.3%)
II	9 (36%)	6 (23%)
III	14 (56%)	17 (65.3%)
**Neuroimaging classification**		
Predominant white matter injury	17 (68.0%)	24 (92.3%)
Predominant grey matter injury	4 (16.0%)	0
Miscellaneous	3 (12.0%)	1 (3.8%)
Normal	1 (4.0%)	1 (3.8%)
**More Affected Extremity**		
Right	12 (48%)	17 (65.4%)
Left	13 (52%)	9 (34.6%)

Table 1 summarizes the demographic and clinical characteristics of participants assigned to remote ischemic conditioning (RIC) and sham control groups at baseline. Variables include age, sex, ethnicity, race, cerebral palsy (CP) severity (type and [Manual Ability Classification System (MACS)] levels), and neuroimaging-based functional classification, and side of more affected extremity. Data are presented as mean (standard deviation) and range for continuous variables, and as frequency (percentage) for categorical variables. Race and ethnicity were reported by caregivers. “Unknown/not reported” indicates that race was not specified. Severity of cerebral palsy was classified according to clinical motor phenotype and *MACS* levels. Neuroimaging classification was based on conventional MRI and categorized as predominant white matter injury, predominant grey matter injury, miscellaneous findings, or normal appearance. Conditioning was performed on the more affected extremity. Children with quadriplegia had superimposed unilateral CP.

## Data Availability

The raw data supporting the conclusions of this article will be made available by the authors on request.
